# Psychological markers associated with ADHD symptom-risk classification among adolescents in Uzbekistan: a school-based cross-sectional study

**DOI:** 10.1186/s40359-026-05545-8

**Published:** 2026-09-07

**Authors:** Dilnoza Akhat kizi Tursunova, Sanjar Boturovich Rakhimmirzaev, Dilbar Gafurdjanovna Mukhamedova

**Affiliations:** Faculty of Social Sciences, Department of Psychology, National University of Uzbekistan named after Mirzo Ulugbek, Tashkent, Uzbekistan

**Keywords:** ADHD symptom risk, Adolescence, Uzbekistan, School-based screening, Multi-informant assessment, Coping, Perceived social support, Self-regulation

## Abstract

**Background:**

School-based screening can identify adolescents who show elevated attention-deficit/hyperactivity disorder (ADHD) symptom risk and may benefit from further psychological assessment or support. However, screening studies are difficult to interpret when risk classification procedures, informant sources, and analytic workflows are not fully described. This study examined psychological markers associated with high ADHD symptom-risk classification among adolescents in Uzbekistan, an underrepresented Central Asian school context.

**Methods:**

A cross-sectional school-based study was conducted in three state general education schools in Tashkent, Uzbekistan. The final analytic sample included 1,512 adolescents aged 12–14 years in grades 7–8. ADHD symptom risk was classified using a study-specific multi-informant screening procedure based on adolescent, parent/legal guardian, and teacher forms. The classification was used for research screening only and was not a clinical ADHD diagnosis. Adolescents completed self-report measures of emotional intelligence, self-regulation, coping strategies, and perceived social support. Descriptive statistics, χ² tests, transparent candidate-variable comparisons, and binary logistic regression were used. The primary regression compared adolescents currently classified as high risk with those classified as medium risk.

**Results:**

Low ADHD symptom risk was identified in 1,152 adolescents (76.19%), medium risk in 255 adolescents (16.87%), and high risk in 105 adolescents (6.94%; 95% CI 5.77–8.34). Risk level was not significantly associated with sex (χ²(2) = 0.90, *p* = 0.636, Cramer’s V = 0.024) or age (χ²(4) = 2.04, *p* = 0.729, Cramer’s V = 0.026). In the high-versus-medium adjusted association model, escape–avoidance coping (OR = 4.47; 95% CI 3.25–6.14) and perceived significant-other support (OR = 2.62; 95% CI 1.81–3.80) were independently associated with high-risk classification, whereas age and sex were not. Model diagnostics are reported descriptively and should not be interpreted as external validation of a clinical prediction tool.

**Conclusions:**

Among adolescents already showing elevated ADHD symptom risk, high-risk classification was mainly distinguished from medium-risk classification by greater escape–avoidance coping and greater perceived support from a significant other. The findings should be interpreted as cross-sectional associations, not as evidence of causal progression or clinical diagnosis. The results suggest possible targets for future school-based psychological support, while also highlighting the need for more detailed item-level, informant-specific, and school-context data in subsequent studies.

**Supplementary Information:**

The online version contains supplementary material available at https://doi.org/10.1186/s40359-026-05545-8.

## Background

Attention-deficit/hyperactivity disorder (ADHD) is a neurodevelopmental condition characterized by persistent patterns of inattention, hyperactivity, and impulsivity that may impair functioning across educational, family, and peer contexts [1, 2]. Prevalence estimates vary according to diagnostic criteria, cultural context, and informant source, but international evidence consistently shows that ADHD-related symptoms are common and consequential during childhood and adolescence [3–5]. In adolescence, overt motor activity may become less salient while difficulties in attention, organization, self-regulation, and emotional control often remain visible in school and family life [6–9].

The present study focuses on ADHD symptom risk rather than clinical diagnosis. This distinction is important because school-based screening procedures can identify adolescents who may benefit from further observation or support, but they cannot replace a diagnostic assessment conducted according to DSM or ICD standards. Screening classifications are therefore best interpreted as indicators of elevated symptom burden or need for further assessment rather than as estimates of clinically diagnosed ADHD [10–13].

Psychological functioning around ADHD symptom risk is multidimensional. Previous research has linked ADHD symptoms with emotion dysregulation, executive and self-regulatory difficulties, coping problems, social impairment, and support needs [6–9, 14–16]. Adolescents who show elevated ADHD symptom risk may differ not only in attentional and behavioral symptoms, but also in how they manage stress, regulate activity, and perceive interpersonal support. These psychological characteristics are particularly relevant for prevention-oriented school psychology because many of them may be addressed through targeted psychological support.

Evidence from Central Asian school contexts remains limited. Recent adolescent mental health research from Uzbekistan has emphasized that empirical school-based mental health data from Central Asia are scarce and that Uzbekistan remains underrepresented in international adolescent mental health literature [40]. The current study contributes school-based data from Tashkent, Uzbekistan, while avoiding the unsupported claim that this is the first study in the region.

The primary analytic question was: among adolescents already showing elevated ADHD symptom risk, which adolescent-reported psychological characteristics distinguish those currently classified as high risk from those currently classified as medium risk? The study was not intended to develop or validate a clinical prediction tool. Rather, it aimed to identify psychological markers associated with high symptom-risk classification that could inform hypotheses for future school-based support and prevention studies.

## Methods

### Study design and setting

This was a cross-sectional school-based study conducted in Tashkent, Uzbekistan, between spring 2025 and January 2026. The study was carried out in three state general education schools. Students were recruited from grades 7–8. Schools participated through cooperation with school administrations and university-facilitated research coordination. The schools were state, general education institutions rather than clinical or specialized ADHD services; therefore, the sample should be interpreted as a school-based sample rather than a clinical sample.

### Participants and participant flow

A total of 1,533 questionnaire packets were returned during data collection. Twenty-one packets were excluded because of incomplete or incorrect completion. The final de-identified analytic sample included 1,512 adolescents aged 12–14 years. No active refusals were recorded by the participating school administrations after the study aims and voluntary nature of participation were explained. Because school-level eligibility denominators and class-level identifiers were not retained in the de-identified analytic dataset, a formal school-population response rate and school/class clustering analyses could not be computed. This limitation is reported explicitly.

The sample characteristics and ADHD symptom-risk distribution are shown in Table 1.

**Table 1:** Participant flow, sample characteristics, and ADHD symptom-risk distribution

Variable	Value	Notes
Schools	state general education schools in Tashkent, Uzbekistan	
Grades	7–8	
Recruitment period	Spring 2025 to January 2026	
Questionnaire packets returned	1,533	
Excluded packets	21	Incorrect or incomplete completion
Final analytic sample	1,512	Complete de-identified analytic records
Girls	654	43.25%
Boys	858	56.75%
Age 12 years	753	49.80%
Age 13 years	376	24.87%
Age 14 years	383	25.33%
Low ADHD symptom risk	1152	76.19% (95% CI 73.98–78.27)
Medium ADHD symptom risk	255	16.87% (95% CI 15.06–18.84)
High ADHD symptom risk	105	6.94% (95% CI 5.77–8.34)

### ADHD symptom-risk screening and classification

ADHD symptom risk was assessed using a study-specific school screening procedure informed by ADHD symptom domains and by the logic of multi-informant behavioral rating. The procedure should not be described as a standard clinical SNAP-IV administration. It used separate content-aligned forms for adolescents, parents/legal guardians, and teachers. Each form included 15 yes/no items describing attentional, hyperactivity/impulsivity, and related behavioral self-control manifestations observed over the previous six months. The adolescent form was phrased as self-report; the parent and teacher forms were phrased as observer reports for home and classroom contexts. English translations of the screening forms and the scoring logic are provided in Additional file 1.

For each form, endorsed symptoms were coded as 1 and non-endorsed symptoms as 0. During primary data processing, the three informant forms were linked by study code. A complete ADHD screening packet required the adolescent, parent/legal guardian, and teacher forms; incomplete packets were excluded. For the integrated screening score, a symptom indicator was counted as present when the corresponding behavioral indicator was supported by at least two of the three informant sources. No single informant was given greater weight. If an indicator was endorsed by only one source, it was not counted in the integrated score. This two-of-three rule was used to reduce reliance on a single rater while recognizing that cross-informant agreement for ADHD-related behavior may be imperfect.

Integrated inattention and hyperactivity/impulsivity domain scores were retained in the analytic file together with an integrated total ADHD symptom-risk score. The total integrated score ranged from 0 to 15. Risk categories were assigned a priori for screening research purposes as follows: 0–3 = low ADHD symptom risk; 4–7 = medium ADHD symptom risk; 8–15 = high ADHD symptom risk. These categories were used to describe screening risk and should not be interpreted as DSM-5 ADHD diagnoses. The de-identified analytic file used for the current revision did not retain the original item-level or informant-specific responses; therefore, informant agreement statistics could not be recalculated. This is acknowledged as a limitation.

The ADHD symptom-risk classification procedure is summarized in Table 2.

**Table 2:** Reproducible ADHD symptom-risk classification procedure

Component	Procedure
Informants	Adolescent self-report, parent/legal guardian report, teacher report
Forms	Separate 15-item yes/no forms, phrased for the relevant informant; content aligned to attentional, hyperactivity/impulsivity, and behavioral self-control indicators
Time frame	Previous six months
Item coding	Endorsed symptom = 1; not endorsed = 0
Integration rule	Symptom counted in the integrated score when at least two of three informants supported the corresponding behavioral indicator
Missing informant data	Incomplete packets were excluded before analysis
Risk categories	Low = 0–3; medium = 4–7; high = 8–15 on the integrated total screening score
Interpretation	Screening risk classification only; not a clinical ADHD diagnosis

### Assessment battery

Adolescents completed the psychological assessment battery using Russian or Uzbek versions of the instruments. Where Uzbek versions were required, items were translated and reviewed by bilingual members of the research team for semantic clarity and age appropriateness. Formal independent back-translation and local psychometric validation were not conducted for all instruments; this is reported as a limitation. The multi-informant ADHD screening forms were used only to classify ADHD symptom risk; the psychological markers examined in the regression analyses were adolescent self-report variables.

The assessment battery and current-sample data availability are shown in Table 3.

**Table 3:** Assessment battery, language/adaptation status, and reliability data availability

Construct	Instrument	Informant	Scales / scoring	Language	Reliability/ data status
ADHD symptom-risk screening	Study-specific multi-informant screening checklist informed by ADHD symptom domains and the logic of SNAP-IV	Adolescent, parent/legal guardian, teacher	15 yes/no items per form; integrated score 0–15; low 0–3, medium 4–7, high ≥ 8	Russian and Uzbek forms; bilingual administration	Item-level and informant-specific responses not retained in the de-identified analytic file; current-sample α could not be calculated.
Emotional intelligence	Hall Emotional Intelligence Test	Adolescent	5 subscales; 30 items in the original form; aggregate subscale scores used	Russian/Uzbek forms	Only aggregate subscale scores were available; item-level α not calculable.
Self-regulation	Style of Self-Regulation of Children’s Behavior (SSPD1-M; Morosanova & Borgoedova)	Adolescent	Planning, modeling, programming, result evaluation, flexibility, autonomy, responsibility	Russian/Uzbek forms	Published instrument for students aged 10–16; item-level α not calculable in current analytic file.
Coping	Ways of Coping Questionnaire (Lazarus & Folkman)	Adolescent	Eight coping scales	Russian/Uzbek forms	Aggregate subscale scores available; item-level α not calculable.
Perceived social support	Multidimensional Scale of Perceived Social Support (MSPSS)	Adolescent	Family, friends, significant other, total/mean support	Russian/Uzbek forms	Aggregate subscale scores available; item-level α not calculable.

### Statistical analysis

Analyses were performed using Python 3.11 with statsmodels, scipy, and scikit-learn. Descriptive statistics were used to summarize participant flow, demographic variables, and ADHD symptom-risk groups. χ² tests and Cramer’s V were used to examine associations between risk group and sex or age.

The primary multivariable analysis was an association model, not a clinical prediction model. It examined which adolescent-reported psychological characteristics were independently associated with high ADHD symptom-risk classification among adolescents already showing elevated symptom risk. Therefore, the primary comparison was high risk versus medium risk. The low-risk group was not included in the primary regression because the question was not whether high-risk adolescents differ from the general low-risk group, but which psychological markers distinguish high from medium risk within the elevated-risk range. Sensitivity analyses using high versus non-high and high versus low comparisons are reported descriptively.

Candidate variables included emotional intelligence subscales, self-regulation subscales, eight coping scales, perceived social support subscales, age, and sex. Core ADHD symptom scores were not included as predictors because they were used to construct the risk classification. Candidate variables were first examined using descriptive statistics and univariable standardized logistic models. The final adjusted model retained age and sex as covariates and included escape-avoidance and perceived significant-other support because these showed the strongest and most interpretable adjusted associations with high-risk status while avoiding direct use of ADHD symptom indicators. A full candidate-variable table is provided in Additional file 1.

Continuous psychological predictors in the final model were standardized within the high-plus-medium risk analysis sample. The reported odds ratios for escape-avoidance and significant-other support therefore correspond to a one-standard-deviation increase in the respective predictor. Sex was coded as boys versus girls, with girls as the reference category. Multicollinearity was evaluated using variance inflation factors (VIF). Model diagnostics included apparent AUC, five-fold outcome-stratified cross-validated AUC for the fixed model, Brier score, and the Hosmer-Lemeshow test. These diagnostics are reported to describe the fitted association model and should not be interpreted as external validation of a clinical prediction tool.

## Results

### Sample characteristics and risk distribution

The final analytic sample included 1,512 adolescents. Girls represented 654 participants (43.25%) and boys represented 858 participants (56.75%). Participants were 12 years old (*n* = 753, 49.80%), 13 years old (*n* = 376, 24.87%), or 14 years old (*n* = 383, 25.33%).

High ADHD symptom risk was identified in 105 adolescents (6.94%; 95% CI 5.77–8.34). Medium risk was identified in 255 adolescents (16.87%), and low risk in 1,152 adolescents (76.19%). ADHD symptom-risk group was not significantly associated with sex (χ²(2) = 0.90, *p* = 0.636, Cramer’s V = 0.024) or age (χ²(4) = 2.04, *p* = 0.729, Cramer’s V = 0.026). These figures describe screening-risk categories, not the prevalence of clinically diagnosed ADHD.

### Psychological markers considered in the analysis

To reduce selective reporting, all psychological variables considered during model development are summarized in Additional file 1. Several coping and perceived support variables showed univariable associations with high-risk status. Emotional intelligence and self-regulation variables were assessed as part of the broader theoretical battery, but they were not retained in the final adjusted model after the candidate-variable review and multivariable specification. These null or non-retained variables are nevertheless reported in Additional file 1.

### Final adjusted association model

The final model compared adolescents classified as high risk (*n* = 105) with those classified as medium risk (*n* = 255). The final binary logistic regression coefficients are shown in Table 4.

**Table 4:** Final adjusted logistic association model for high versus medium ADHD symptom-risk classification

Variable	B	SE	Adjusted OR	95% CI	*p*
Age, years	-0.038	0.186	0.96	0.67–1.38	= 0.837
Sex, male vs. female	-0.198	0.309	0.82	0.45–1.50	= 0.521
Escape-avoidance, per 1 SD	1.497	0.162	4.47	3.25–6.14	< 0.001
Significant-other support, per 1 SD	0.964	0.189	2.62	1.81–3.80	< 0.001

Escape–avoidance coping was independently associated with high-risk classification. A one-standard-deviation increase in escape–avoidance was associated with higher odds of being classified as high risk rather than medium risk. Perceived support from a significant other was also independently associated with high-risk classification. Age and sex were not independently associated with high-risk classification in the final model.

### Model diagnostics and sensitivity analyses

Model performance and calibration indices are summarized in Table 5.

**Table 5:** Model diagnostics for the final association model

Index	Result	Interpretation
Analysis sample	360 adolescents	High risk *n* = 105; medium risk *n* = 255
Likelihood-ratio test	χ²(4) = 161.16; *p* < 0.001	Final association model statistically improved fit over intercept-only model
AUC, apparent	0.885 (bootstrap 95% CI 0.846–0.920)	Discrimination estimate for the fitted model, not an externally validated prediction tool
Five-fold cross-validated AUC	0.875	Fixed model refitted in outcome-stratified folds; feature selection was not repeated within each fold
Brier score	0.122	Overall prediction error for the fitted probabilities
Hosmer-Lemeshow	χ²(8) = 7.92; *p* = 0.441	No evidence of poor calibration; test interpreted cautiously
Maximum VIF	2.20	No evidence of substantial multicollinearity among final model covariates

Sensitivity analyses are reported descriptively because the primary research question concerned high versus medium risk among adolescents already showing elevated symptom risk. In the high-versus-non-high and high-versus-low comparisons, escape-avoidance and perceived significant-other support remained associated with high-risk classification. However, these comparisons should not be interpreted as stronger evidence of prediction because they answer a different and easier group-separation question. (Table 6).

**Table 6:** Sensitivity analyses using alternative comparison groups

Analysis	Variable	OR	95% CI	*p*	Model AUC
Primary: high vs. medium	Escape-avoidance, per 1 SD	4.47	3.25–6.14	< 0.001	AUC 0.885
Primary: high vs. medium	Significant-other support, per 1 SD	2.62	1.81–3.80	< 0.001	AUC 0.885
Sensitivity: high vs. non-high	Escape-avoidance, per 1 SD	3.98	3.18–4.98	< 0.001	AUC 0.953
Sensitivity: high vs. non-high	Significant-other support, per 1 SD	4.16	3.08–5.64	< 0.001	AUC 0.953
Sensitivity: high vs. low	Escape-avoidance, per 1 SD	5.46	4.03–7.39	< 0.001	AUC 0.969
Sensitivity: high vs. low	Significant-other support, per 1 SD	5.72	4.02–8.13	< 0.001	AUC 0.969

## Discussion

### Principal findings

This study examined psychological markers associated with ADHD symptom-risk classification in a school-based adolescent sample from Uzbekistan. The study identified a relatively small but practically important high-risk screening group within a large school sample. This pattern is expected in school-based screening: high symptom-risk cases represent a minority of students, and a broad screening sample is required to identify a sufficiently informative high-risk subgroup.

Among adolescents already showing elevated ADHD symptom risk, high-risk classification was mainly distinguished from medium-risk classification by higher escape-avoidance coping and higher perceived support from a significant other. The finding for escape-avoidance is consistent with the possibility that adolescents with greater attentional and self-regulatory difficulties may be more likely to disengage from demanding situations. However, the cross-sectional design does not establish whether avoidance contributes to elevated symptom risk, whether elevated symptom risk contributes to avoidance, or whether the association is bidirectional.

The finding for significant-other support should also be interpreted cautiously. The MSPSS assesses perceived support, not dependency, interpersonal scaffolding, or external regulation. Therefore, the result means that adolescents classified as high risk reported greater perceived support from a significant other. Several explanations are possible: adolescents with greater difficulties may receive more support, may perceive available support differently, or may report a salient non-family/non-friend support figure because of school or family circumstances. The present study does not demonstrate the mechanism behind this association.

### Interpretation in relation to previous research

Previous studies have linked ADHD symptoms with emotion dysregulation, executive and self-regulatory difficulties, social difficulties, and service needs [6–17]. The present findings are consistent with that broader literature but refine the interpretation for a school-based screening context. Rather than suggesting a comprehensive independent psychological profile, the final adjusted model indicates that the high-versus-medium distinction was mainly characterized by escape-avoidance coping and perceived significant-other support after adjustment for age and sex.

The results also support the value of multi-informant school screening. Parent and teacher reports provide information about behavior across home and classroom contexts, while adolescent self-report captures subjective experience. At the same time, the current de-identified analytic file did not retain informant-specific item-level data. Therefore, the study cannot report agreement statistics among adolescents, parents, and teachers, and this limits conclusions about the contribution of each informant.

### Implications for school-based support

The findings should be treated as hypotheses for future school-based psychological support, not as tested intervention recommendations. Adolescents with elevated ADHD symptom risk may benefit from further psychological assessment, monitoring of avoidance-based coping, and attention to the structure and meaning of perceived interpersonal support. The data do not show that changing escape-avoidance or significant-other support will reduce ADHD symptom risk. Future intervention studies should test whether targeted support around coping, task engagement, and developmentally appropriate support relationships improves functioning in adolescents identified through school-based screening.

### Strengths and limitations

Strengths of the study include the large school-based sample, the focus on adolescents aged 12–14 years, the inclusion of adolescent, parent, and teacher information in ADHD symptom-risk classification, and the contribution of data from Uzbekistan. The study also explicitly distinguishes screening risk from clinical diagnosis.

Several limitations are important. First, ADHD symptom risk was classified through a school-based screening procedure and not through clinical diagnostic interview. The reported 6.94% should not be interpreted as prevalence of clinically diagnosed ADHD. Second, the de-identified analytic file did not retain item-level responses or informant-specific ADHD records, preventing calculation of current-sample Cronbach’s alpha or omega, informant agreement statistics, and parent/teacher-only sensitivity analyses. Published reliability evidence is useful background information, but it does not replace reliability estimates from the current sample. Third, school and classroom identifiers were not available in the de-identified analytic dataset; therefore, clustering within schools or classes could not be modeled. Fourth, detailed school-level socioeconomic characteristics, class sizes, student-to-teacher ratios, and family-level socioeconomic indicators were not available. Residual confounding by anxiety, depressive symptoms, sleep problems, academic performance, learning difficulties, family characteristics, medication use, and other neurodevelopmental or behavioral conditions remains possible. Fifth, formal independent back-translation and local validation procedures were not available for all instruments, even though Russian and Uzbek versions were used and reviewed by the research team. Sixth, the cross-sectional design prevents conclusions about temporal progression, causality, or prediction of future ADHD symptom risk.

## Conclusions

In a school-based adolescent sample from Uzbekistan, high ADHD symptom-risk classification was identified in 6.94% of participants. Among adolescents already showing elevated symptom risk, high-risk classification was independently associated with greater escape-avoidance coping and greater perceived support from a significant other. These findings identify cross-sectional psychological markers that may inform hypotheses for school-based screening and targeted support. They do not establish clinical diagnosis, causal pathways, or tested intervention recommendations. Future research should replicate these findings using item-level data, informant-specific records, school/class identifiers, local psychometric validation, and longitudinal designs.

## Supplementary Information

Below is the link to the electronic supplementary material.

Supplementary Material 1

## Data Availability

The datasets used and/or analyzed during the current study are available from the corresponding author upon reasonable request, subject to institutional and ethical restrictions.

## Abbreviations

ADHD: attention-deficit/hyperactivity disorder
AUC: area under the receiver operating characteristic curve
CI: confidence interval
MSPSS: Multidimensional Scale of Perceived Social Support
OR: odds ratio
SSPD1-M: Style of Self-Regulation of Children’s Behavior
VIF: variance inflation factor
