# Supplementary material for: Psychological markers associated with ADHD symptom-risk classification among adolescents in Uzbekistan: a school-based cross-sectional study
Source: BMC Psychol. 2026 Sep 7;14:1643. doi: 10.1186/s40359-026-05545-8 (PMC13760859; doi:10.1186/s40359-026-05545-8)
Supplement: Supplementary file 1 [file 40359_2026_5545_MOESM1_ESM.docx]

**Additional file 1. Methodological appendix**

Manuscript: Psychological markers associated with ADHD symptom-risk classification among adolescents in Uzbekistan: a school-based cross-sectional study

This appendix provides supplementary information requested during peer review: participant flow, ADHD screening forms and scoring algorithm, assessment battery provenance, candidate-variable transparency, model diagnostics, and STROBE-oriented reporting notes.

# A. Participant flow and school context

**Table S1. Participant flow and school context**

| **Item** | **Description** |
| --- | --- |
| Setting | Three state general education schools in Tashkent, Uzbekistan |
| School identifiers | Not reported to preserve institutional confidentiality; school/class identifiers were not retained in the de-identified analytic dataset |
| Grades | 7-8 |
| Recruitment period | Spring 2025 to January 2026 |
| Recruitment route | Schools were recruited through cooperation between university administration/research team and school administrations |
| Questionnaire packets returned | 1,533 |
| Excluded packets | 21 due to incomplete or incorrect completion |
| Final analytic sample | 1,512 |
| School-level socioeconomic/resource indicators | Not available in the de-identified analytic dataset |
| Clustering | School/class identifiers unavailable; clustering could not be modeled |

# B. ADHD symptom-risk screening forms and scoring

The study used content-aligned adolescent, parent/legal guardian, and teacher screening forms. The forms were designed for school-based screening and were not used to establish clinical diagnosis. The English versions below are translations prepared for peer-review transparency.

## B1. Adolescent self-report form

**Table S2. Adolescent ADHD symptom-risk screening form**

| **Item** | **Adolescent self-report item** |
| --- | --- |
| 1 | Difficulty focusing on details when completing assignments or other activities. |
| 2 | Frequent careless mistakes in school assignments or other work. |
| 3 | Difficulty sustaining attention during tasks or games. |
| 4 | Often does not seem to listen when spoken to directly. |
| 5 | Often does not follow instructions through or does not complete started tasks. |
| 6 | Difficulty organizing activities and time. |
| 7 | Avoids tasks requiring prolonged mental effort. |
| 8 | Often loses things needed for tasks, such as books, pens, or materials. |
| 9 | Easily distracted by external stimuli. |
| 10 | Often forgets daily activities. |
| 11 | Moves more than needed when the situation requires calm behavior. |
| 12 | Difficulty remaining seated in class or other situations where sitting is required. |
| 13 | Often feels overly active or as if 'driven by a motor'. |
| 14 | Often talks more than needed. |
| 15 | Difficulty waiting for a turn or often interrupts others. |

## B2. Parent/legal guardian report form

**Table S3. Parent/legal guardian ADHD symptom-risk screening form**

| **Item** | **Parent/legal guardian report item** |
| --- | --- |
| 1 | Often loses things needed for school or home. |
| 2 | Does not listen when addressed directly. |
| 3 | Does not complete started activities. |
| 4 | Easily distracted during activities. |
| 5 | Often forgets to carry out instructions or requests. |
| 6 | Cannot sit calmly at a table for a prolonged period. |
| 7 | Often gets up when expected to remain seated. |
| 8 | Shows excessive motor activity. |
| 9 | Often talks more than needed. |
| 10 | Often interrupts others. |
| 11 | Becomes irritated or angry easily. |
| 12 | Often does not complete assignments or tasks. |
| 13 | Acts without thinking. |
| 14 | Shows disobedience toward parents/caregivers. |
| 15 | Has conflicts with siblings or other children. |

## B3. Teacher report form

**Table S4. Teacher ADHD symptom-risk screening form**

| **Item** | **Teacher report item** |
| --- | --- |
| 1 | Listens attentively to explanations in class. |
| 2 | Often becomes distracted during lessons. |
| 3 | Completes school assignments. |
| 4 | Disturbs other students. |
| 5 | Gets up from the seat without permission. |
| 6 | Shows excessive talkativeness in class. |
| 7 | Often interrupts the teacher. |
| 8 | Has difficulty completing written assignments. |
| 9 | Becomes irritated over minor issues. |
| 10 | Argues with classmates. |
| 11 | Violates classroom discipline. |
| 12 | Often acts without thinking. |
| 13 | Completes tasks on time. |
| 14 | Shows restlessness during long activities. |
| 15 | Has conflicts with teachers. |

## B4. Integrated scoring and classification rule

**Table S5. Integrated ADHD symptom-risk scoring procedure**

| **Scoring element** | **Procedure** |
| --- | --- |
| Form response coding | Yes / symptom present = 1; No / symptom absent = 0. Reverse-phrased observer items were coded so that higher values indicated greater symptom expression or behavioral concern. |
| Informant linkage | Adolescent, parent/legal guardian, and teacher forms were linked by study code during data collection and scoring. |
| Complete packet rule | Packets were included only when the forms required for integrated screening were complete; incomplete/incorrect packets were excluded before de-identification. |
| Integrated item rule | A symptom indicator was counted as present in the integrated score when at least two of the three informants supported the corresponding behavioral indicator. |
| Informant weighting | No informant was assigned greater weight in the integrated coding rule. |
| Risk score | Integrated total ADHD symptom-risk score retained in the analytic file ranged from 0 to 15. |
| Risk categories | Low = 0-3; medium = 4-7; high = 8-15. |
| Interpretation | Categories indicate screening risk, not clinical ADHD diagnosis. |

# C. Assessment battery, language, and current-sample reliability status

The table below clarifies instrument provenance, language/adaptation status, and why current-sample internal consistency could not be computed from the de-identified analytic file. This appendix avoids reporting Cronbach's alpha across heterogeneous subscales because such coefficients would not answer the reviewers' psychometric question.

**Table S6. Assessment battery and data availability for reliability estimation**

| **Construct** | **Instrument** | **Items/scales** | **Language/adaptation** | **Data available in analytic file** | **Reliability status** |
| --- | --- | --- | --- | --- | --- |
| ADHD symptom risk | Study-specific multi-informant screening form informed by ADHD symptom domains | 15 yes/no items per informant form; integrated domain and total scores | Russian and Uzbek forms | Integrated inattention, hyperactivity/impulsivity, and total score | Item-level and informant-specific responses not retained; current-sample α/kappa not computable |
| Emotional intelligence | Hall Emotional Intelligence Test | 30 items; 5 subscales | Russian and Uzbek forms | Subscale scores and total score | Item-level responses not retained; current-sample α not computable |
| Self-regulation | SSPD1-M by Morosanova and Borgoedova | 7 regulatory subscales | Russian and Uzbek forms | Subscale scores | Item-level responses not retained; current-sample α not computable |
| Coping | Ways of Coping Questionnaire | 8 coping scales | Russian and Uzbek forms | Subscale scores | Item-level responses not retained; current-sample α not computable |
| Perceived social support | MSPSS | Family, friends, significant other, total/mean support | Russian and Uzbek forms | Subscale scores and mean score | Item-level responses not retained; current-sample α not computable |

# D. Candidate-variable transparency

The following table reports all adolescent-reported psychological variables considered in the high-versus-medium risk analysis. Medium-risk adolescents are the reference comparison group for descriptive statistics. Odds ratios are univariable, based on standardized continuous predictors, and should not be compared directly with adjusted odds ratios in the main regression model.

**Table S7. Candidate psychological variables considered in model development**

| **Variable** | **Medium mean (SD)** | **High mean (SD)** | **SMD** | **Univariable OR** | **95% CI** | **p** | **BH q** |
| --- | --- | --- | --- | --- | --- | --- | --- |
| Accepting responsibility | 6.91 (5.08) | 10.59 (5.43) | 0.71 | 1.91 | 1.52-2.39 | < .001 | < .001 |
| Autonomy | 4.65 (1.23) | 4.67 (1.16) | 0.01 | 1.01 | 0.80-1.27 | = .933 | = .933 |
| Confrontive coping | 10.93 (4.42) | 12.13 (3.98) | 0.28 | 1.32 | 1.05-1.65 | = .017 | = .030 |
| Coping mean | 10.10 (1.78) | 13.12 (1.57) | 1.75 | 11.20 | 6.66-18.85 | < .001 | < .001 |
| Distancing | 10.31 (3.22) | 12.82 (4.61) | 0.68 | 1.93 | 1.52-2.44 | < .001 | < .001 |
| Emotional awareness | 6.81 (2.76) | 7.09 (2.95) | 0.10 | 1.10 | 0.88-1.37 | = .401 | = .497 |
| Emotional intelligence total | 38.84 (7.50) | 37.24 (8.16) | -0.21 | 0.81 | 0.64-1.02 | = .074 | = .101 |
| Empathy | 6.22 (3.21) | 6.29 (3.98) | 0.02 | 1.02 | 0.81-1.28 | = .876 | = .931 |
| Escape–avoidance | 6.91 (5.08) | 14.88 (4.67) | 1.60 | 4.27 | 3.19-5.70 | < .001 | < .001 |
| Flexibility | 4.78 (0.98) | 4.99 (0.96) | 0.21 | 1.25 | 0.98-1.59 | = .070 | = .101 |
| MSPSS mean | 3.75 (0.91) | 4.52 (0.76) | 0.88 | 2.72 | 2.02-3.68 | < .001 | < .001 |
| Managing own emotions | 6.60 (2.70) | 6.55 (3.24) | -0.02 | 0.98 | 0.78-1.24 | = .895 | = .931 |
| Modeling | 4.68 (1.25) | 4.63 (1.13) | -0.04 | 0.96 | 0.76-1.20 | = .702 | = .830 |
| Perceived family support | 3.12 (1.28) | 3.56 (1.27) | 0.34 | 1.40 | 1.12-1.75 | = .004 | = .008 |
| Perceived friend support | 4.00 (1.44) | 4.59 (1.33) | 0.42 | 1.54 | 1.21-1.95 | < .001 | = .001 |
| Perceived significant-other support | 4.14 (1.86) | 5.41 (1.21) | 0.75 | 2.38 | 1.77-3.21 | < .001 | < .001 |
| Planful problem solving | 10.71 (4.10) | 12.42 (5.07) | 0.39 | 1.47 | 1.16-1.85 | = .001 | = .003 |
| Planning | 5.25 (0.87) | 5.22 (0.80) | -0.04 | 0.96 | 0.77-1.21 | = .745 | = .842 |
| Positive reappraisal | 10.16 (5.04) | 14.65 (5.04) | 0.89 | 2.52 | 1.92-3.29 | < .001 | < .001 |
| Programming | 4.82 (1.09) | 4.60 (1.00) | -0.20 | 0.82 | 0.66-1.03 | = .083 | = .108 |
| Recognizing others’ emotions | 10.77 (4.50) | 9.69 (4.71) | -0.24 | 0.79 | 0.63-0.99 | = .041 | = .067 |
| Responsibility | 4.81 (0.92) | 5.22 (0.64) | 0.48 | 1.79 | 1.34-2.40 | < .001 | < .001 |
| Result evaluation | 4.98 (1.02) | 5.30 (0.82) | 0.33 | 1.44 | 1.11-1.87 | = .006 | = .011 |
| Seeking social support | 10.22 (5.31) | 13.35 (4.71) | 0.61 | 1.85 | 1.45-2.35 | < .001 | < .001 |
| Self-controlling | 12.95 (4.78) | 14.42 (4.00) | 0.32 | 1.39 | 1.10-1.76 | = .006 | = .012 |
| Self-motivation | 8.44 (3.72) | 7.63 (3.93) | -0.21 | 0.80 | 0.63-1.02 | = .066 | = .101 |

# E. Model diagnostics and sensitivity analyses

**Table S8. Sensitivity analyses using alternative comparison groups**

| **Analysis** | **Variable** | **OR** | **95% CI** | **p** | **Model AUC** |
| --- | --- | --- | --- | --- | --- |
| Primary: high vs medium | Escape–avoidance, per 1 SD | 4.47 | 3.25-6.14 | < .001 | AUC 0.885 |
| Primary: high vs medium | Significant-other support, per 1 SD | 2.62 | 1.81-3.80 | < .001 | AUC 0.885 |
| Sensitivity: high vs non-high | Escape–avoidance, per 1 SD | 3.98 | 3.18-4.98 | < .001 | AUC 0.953 |
| Sensitivity: high vs non-high | Significant-other support, per 1 SD | 4.16 | 3.08-5.64 | < .001 | AUC 0.953 |
| Sensitivity: high vs low | Escape–avoidance, per 1 SD | 5.46 | 4.03-7.39 | < .001 | AUC 0.969 |
| Sensitivity: high vs low | Significant-other support, per 1 SD | 5.72 | 4.02-8.13 | < .001 | AUC 0.969 |

The sensitivity analyses support the direction of the two main associations, but the high-versus-non-high and high-versus-low comparisons answer different questions and are easier group-separation tasks than the primary high-versus-medium analysis. Therefore, they are not treated as the main evidence.

# F. STROBE-oriented reporting status

**Table S9. STROBE-oriented reporting status**

| **STROBE item area** | **Where addressed** |
| --- | --- |
| Title/abstract | Study design and cross-sectional nature stated in title and abstract |
| Background/rationale | ADHD symptom risk, school-based screening, and Central Asian context described |
| Objectives | Association-oriented research question stated |
| Study design | Cross-sectional school-based design specified |
| Setting | Three state general education schools in Tashkent; period stated |
| Participants | Participant flow and exclusion count reported; school-level denominators not retained |
| Variables | Outcome, candidate variables, covariates, and risk categories described |
| Data sources/measurement | Instruments, informants, language/adaptation, and data limitations described |
| Bias | Selection bias, shared-rater variance, residual confounding, and clustering limitations acknowledged |
| Study size | Final N and high/medium/low groups reported |
| Quantitative variables | Continuous standardized predictors described; dichotomized marker table removed |
| Statistical methods | χ² tests, logistic association model, diagnostics, sensitivity analyses described |
| Participants/results | Sample characteristics and risk distributions reported |
| Main results | Adjusted ORs and CIs reported |
| Other analyses | Sensitivity analyses and candidate-variable table provided |
| Limitations | Major methodological and data-availability limitations reported |
| Generalizability | Limited to school-based adolescents in one Uzbek context |
| Funding | No specific funding stated |
